# Supplementary material for: Content-rich biological network constructed by mining PubMed abstracts
Source: BMC Bioinformatics. 2004 Oct 8;5:147. doi: 10.1186/1471-2105-5-147 (PMC528731; doi:10.1186/1471-2105-5-147)
Supplement: Additional File 2 — The original results of the above study (non-essential files are deleted to keep the file size under the limit set by BMC bioinformatics). [file 1471-2105-5-147-S2.bz2 › chilibotAdditionalFile2/dip10/554ID10049744E10914/html/IFNAR1_IFNB1.html]

 


 **IFNAR1** and **IFNB1** 
  
Found 357 abstracts in PubMed, retrieved 10.  
 

 What does Google say? 
 PDF only 
| .edu only 

---

**Interactive relationship** (e.g. stimulation, inhibition, etc)

**Inhibitory relationship**- Interferon alpha  [ **IFNAR1** ]  effectively inhibited SARS CoV replication, but with a selectivity index 50 90 times lower than that for interferon beta  [ **IFNB1** ] .  Ref: 12892961 Lancet, 2003
**Neutral relationship**- In murine macrophages, IFN beta  [ **IFNB1** ]  induced TLR3 up regulation required  **IFNAR1** , STAT1, and in part IRF 1, but not the Janus kinase Jak family member Tyk2.  Ref: 12672806 J Biol Chem< MedlineTA>J Biol Chem, 2003

**Non-interactive relationship** (e.g. studied together, co-existance, homology, etc.)

- the 1B 2R1 replicon was sensitive to interferon alpha  [ **IFNAR1** ] , interferon beta  [ **IFNB1** ] , and interferon gamma.  Ref: 12810084 Biochem Biophys Res Commun, 2003
- Interferon alpha  [ **IFNAR1** ]  and Interferon beta  [ **IFNB1** ]  can inhibit recurrence in HCC patients with HCV infection background, though the mechanism is not fully understood.  Ref: 12679900 World J Gastroenterol, 2003
- Other pro inflammatory cytokines representing potential therapeutic targets include interferon beta  [ **IFNB1** ] , interferon alpha  [ **IFNAR1** ] , IL 6, IL 15, IL 17 and IL 18.  Ref: 12769749 Curr Pharm Des, 2003
- To investigate the intra hepatic activation of the IFN system in patients affected by chronic HCV infection in comparison with that observed in a non infectious liver disease such as non alcoholic steatohepatitis, we measured the liver steady state mRNA levels of interferon alpha  [ **IFNAR1** ] , interferon beta  [ **IFNB1** ]  and interferon gamma as well as of IFN related genes IFNAR 1  [ **IFNAR1** ] , STAT1alpha, PKR, 2 5 AS, IRF 1, ICE and IL 18.  Ref: 12794720 J Med Virol< MedlineTA>J Med Virol, 2003
- Considering the increased risk of reactivation of autoimmune phenomena during a six months therapy with interferon alpha  [ **IFNAR1** ] , an intensive combination therapy with interferon beta  [ **IFNB1** ]  and gamma 2 weeks 1x3 MIU nIFN beta Fiblaferon R i.  Ref: 12772056 Z Gastroenterol< MedlineTA>Z Gastroenterol, 2003
